# Extensive Gene Amplification as a Mechanism for Piperacillin-Tazobactam Resistance in Escherichia coli

**DOI:** 10.1128/mBio.00583-18

**Published:** 2018-04-24

**Authors:** Lisa M. Schechter, David P. Creely, Cherilyn D. Garner, Dee Shortridge, Hoan Nguyen, Lei Chen, Blake M. Hanson, Erica Sodergren, George M. Weinstock, W. Michael Dunne, Alex van Belkum, Shana R. Leopold

**Affiliations:** a BioMérieux, Inc., Hazelwood, Missouri, USA; b Jackson Laboratory for Genomic Medicine, Farmington, Connecticut, USA; c BioMérieux, Inc., Durham, North Carolina, USA; d BioMérieux SA, La Balme les Grottes, France; Brigham and Women’s Hospital

**Keywords:** *Escherichia coli*, TEM-1, antibiotic resistance, gene amplification, piperacillin-tazobactam

## Abstract

Although the TEM-1 β-lactamase (Bla_TEM-1_) hydrolyzes penicillins and narrow-spectrum cephalosporins, organisms expressing this enzyme are typically susceptible to β-lactam/β-lactamase inhibitor combinations such as piperacillin-tazobactam (TZP). However, our previous work led to the discovery of 28 clinical isolates of Escherichia coli resistant to TZP that contained only *bla*_TEM-1_. One of these isolates, E. coli 907355, was investigated further in this study. E. coli 907355 exhibited significantly higher β-lactamase activity and Bla_TEM-1_ protein levels when grown in the presence of subinhibitory concentrations of TZP. A corresponding TZP-dependent increase in *bla*_TEM-1_ copy number was also observed, with as many as 113 copies of the gene detected per cell. These results suggest that TZP treatment promotes an increase in *bla*_TEM-1_ gene dosage, allowing Bla_TEM-1_ to reach high enough levels to overcome inactivation by the available tazobactam in the culture. To better understand the nature of the *bla*_TEM-1_ copy number proliferation, whole-genome sequence (WGS) analysis was performed on E. coli 907355 in the absence and presence of TZP. The WGS data revealed that the *bla*_TEM-1_ gene is located in a 10-kb genomic resistance module (GRM) that contains multiple resistance genes and mobile genetic elements. The GRM was found to be tandemly repeated at least 5 times within a p1ESCUM/p1ECUMN-like plasmid when bacteria were grown in the presence of TZP.

## IMPORTANCE

Understanding how bacteria acquire resistance to antibiotics is essential for treating infected patients effectively, as well as preventing the spread of resistant organisms. In this study, a clinical isolate of E. coli was identified that dedicated more than 15% of its genome toward tandem amplification of a ~10-kb resistance module, allowing it to escape antibiotic-mediated killing. Our research is significant in that it provides one possible explanation for clinical isolates that exhibit discordant behavior when tested for antibiotic resistance by different phenotypic methods. Our research also shows that GRM amplification is difficult to detect by short-read WGS technologies. Analysis of raw long-read sequence data was required to confirm GRM amplification as a mechanism of antibiotic resistance.

## INTRODUCTION

β-Lactam antibiotics are an important class of antimicrobial agents that include penicillins, narrow- and extended-spectrum cephalosporins, monobactams, and carbapenems. They act by inhibiting cell wall synthesis and promoting cell lysis upon osmotic shock. There are four main resistance mechanisms that allow bacteria to circumvent the effects of β-lactams: target modification of bacterial penicillin binding proteins (PBPs), upregulation of efflux pumps, alteration of porin expression or function, and β-lactamase enzyme production (1). The combined presence of these mechanisms in individual strains has been documented (2).

β-Lactamases are enzymes that inactivate β-lactam antibiotics by hydrolyzing the β-lactam ring. More than 2,000 unique naturally occurring β-lactamase enzymes have been identified to date (3). The TEM β-lactamases, a group containing more than 200 variants (http://www.lahey.org/Studies/temtable.asp), are all presumably derived from the first identified TEM protein, TEM-1. The Bla_TEM-1_ enzyme hydrolyzes penicillins and narrow-spectrum cephalosporins, placing it in functional classification group 2b (4, 5). According to the molecular classification system, it is a class A serine β-lactamase (6). Although the *bla*_TEM-1_ gene is usually plasmid-borne, it is actively spread via transposition and its insertion into the chromosome has been documented (7–10).

To preserve and extend the utility of β-lactams, some have been used in combination with β-lactmase inhibitors. For example, amoxicillin, ampicillin, and piperacillin are used in combination with the β-lactamase inhibitors clavulanate, sulbactam, and tazobactam, respectively. These classical inhibitors render most organisms that express class A serine β-lactamases susceptible to β-lactams, excluding a few enzymes that are inhibitor resistant, such as Klebsiella pneumoniae carbapenemases (KPCs) (1). However, intensive use of β-lactamase inhibitors has caused the emergence of inhibitor-resistant variants within the TEM and SHV β-lactamase families. In addition, overexpression of inhibitor-sensitive enzymes, such as Bla_TEM-1_, has led to clavulanate, sulbactam, and/or tazobactam resistance (11–20). In some cases, higher Bla_TEM-1_ enzyme levels were attributed to a *Pa/Pb* mutation in the *bla*_TEM-1_ promoter that increased transcription (12, 17, 18, 21). Bla_TEM-1_ hyperproduction resulting from an increase in *bla*_TEM-1_ gene dosage has also been documented (12, 13, 15, 22, 23).

We have identified a number of clinical E. coli isolates that exhibit discordant behavior and heterogeneous resistance in disk diffusion (DD), agar dilution (AD), and broth microdilution (BMD) antibiotic susceptibility testing (AST) assays (24, 25). A PCR- and sequence-based screen for plasmid-borne β-lactamase genes revealed that many of the isolates harbored only *bla*_TEM-1_. In this study, we further characterized one of these clinical isolates, combining phenotypic and genotypic approaches to characterize a unique mechanism of antibiotic resistance.

## RESULTS

Previous work revealed that E. coli clinical isolate 907355 is resistant to piperacillin-tazobactam (TZP; MIC for the combination, >128 µg/ml piperacillin and 4 µg/ml tazobactam [expressed here as ≥128/4 µg/ml]) in BMD tests, yet susceptible (MIC, ≤16/4 µg/ml) by the AD method (24, 25). To further investigate the nature of this discordant behavior, BMD assays were performed with two other β-lactam/β-lactamase inhibitor combinations, ampicillin-sulbactam (SAM) and amoxicillin-clavulanate (AMC). According to Clinical and Laboratory Standards Institute (CLSI) breakpoints (26), E. coli 907355 is resistant to both SAM (MIC, >64/32 µg/ml) and AMC (MIC, 32/16 µg/ml) by BMD (Table 1). Thus, the mechanism underlying E. coli 907355 resistance to TZP likely extends to other β-lactam/β-lactamase inhibitor combinations.

**TABLE 1:** Summary of phenotypic and genotypic characteristics of E. coli 907355

Genotype or phenotype examined	Result
SAM^a^ MIC (µg/ml)	>64/32
AMC^a^ MIC (µg/ml)	32/16
β-Lactamase pI^b^	5.4
*bla*_TEM-1_ promoter	*P3*
OmpF expression^c^	Yes
OmpC expression^c^	Yes
Increased efflux (PAβN)^d^	No

a MICs for SAM (ampicillin-sulbactam) and AMC (amoxicillin-clavulanic acid) were determined in broth microdilution assays.

b β-Lactamase pI was determined by IEF.

c OmpF and OmpC expression was detected by Western immunoblotting.

d Increased efflux was measured in an PAβN inhibitor assay. A ≥4-fold decrease in the TZP MIC in the presence of 50 µM PAβN was considered significant.

**FIG 1:**
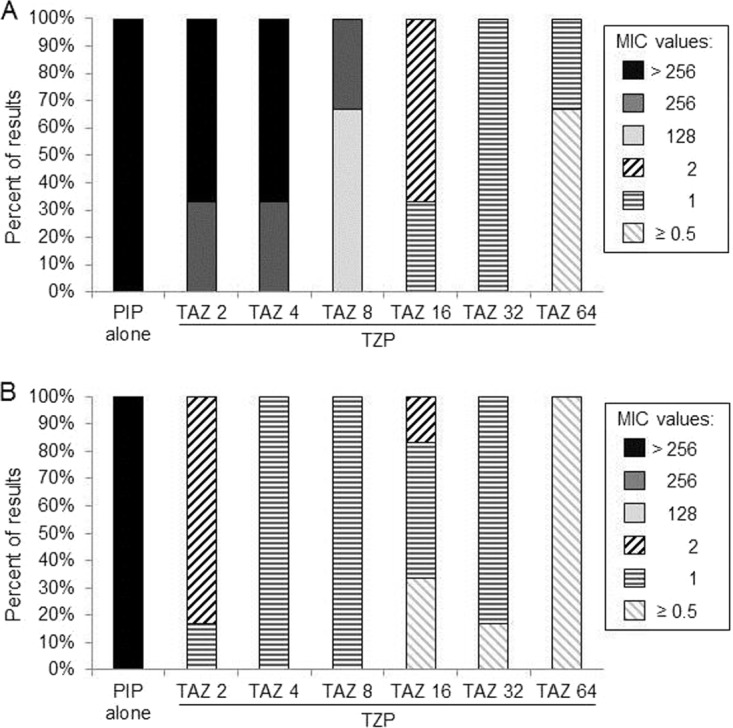
Effect of increasing tazobactam concentrations on E. coli susceptibility to TZP. MICs were determined for 907355 (A) and ATCC 35218 (B) via BMD in the presence of piperacillin (PIP) alone or TZP formulations containing different fixed concentrations (2 to 64 µg/ml) of tazobactam (TAZ). The MIC values in the legend are based on the concentration of piperacillin in the TZP formulation that inhibited growth. The range of MIC values obtained from six experiments is represented within each stacked bar on the graphs.

We next examined whether increasing the concentration of tazobactam within the TZP formulation renders 907355 susceptible to piperacillin in BMD assays. E. coli 907355 exhibited a TZP MIC of ≥128 µg/ml when the tazobactam concentration was between 0 and 8 µg/ml, whereas 16 to 64 µg/ml tazobactam reduced the 907355 TZP MIC to ≤2 µg/ml (Fig. 1A). As a control, a BMD assay was performed with 128 µg/ml tazobactam alone, which did not affect 907355 growth (data not shown). Therefore, the decrease in the 907355 TZP MIC at higher tazobactam concentrations was not due to tazobactam toxicity. The experiment was also performed on E. coli ATCC 35218, a CLSI quality control (QC) organism that expresses Bla_TEM-1_ and is resistant to piperacillin yet susceptible to TZP. The MICs for ATCC 35218 were ≤2 µg/ml at all tazobactam concentrations tested (Fig. 1B), revealing that 907355 requires a much higher concentration of tazobactam than ATCC 35218 for TZP to be effective.

We previously performed PCR assays on E. coli 907355 to detect multiple plasmid-borne β-lactamase genes. The product from the only positive reaction was sequenced, revealing that E. coli 907355 contains *bla*_TEM-1_ (24). At the deduced amino acid sequence level, E. coli 907355 Bla_TEM-1_ is 100% identical to the original Bla_TEM-1_. Therefore, 907355 resistance to TZP, SAM, and AMC is not due to acquisition of inhibitor resistance mutations within the *bla*_TEM-1_ gene. Further analysis of 907355 by isoelectric focusing (IEF) confirmed that the isolate expresses only the Bla_TEM-1_ β-lactamase, which has a pI of 5.4 (Table 1). In addition, the 907355 *bla*_TEM-1_ gene has a wild-type *P3* promoter (−35 to +1) region (Table 1). In a previous study, the *P3* promoter directed relatively low levels of *bla*_TEM_ expression compared to the *Pa/Pb*, *P4*, and *P5* promoters (21). Therefore, sequence variations in the RNA polymerase binding site are likely not the primary cause of β-lactam–β-lactamase inhibitor resistance in E. coli 907355.

**FIG 2:**
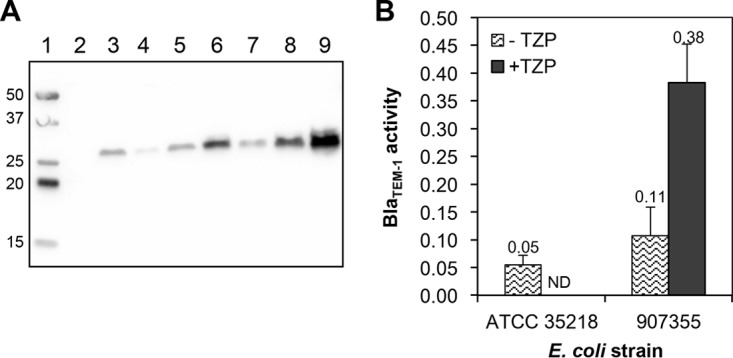
Effects of TZP on E. coli 907355 Bla_TEM-1_ levels and β-lactamase enzyme activity. (A) Detection of Bla_TEM-1_ in E. coli ATCC 25922 (lane 2), ATCC 35218 (lanes 3 to 5), and 907355 (lanes 6 to 9) by Western immunoblotting. Strains were cultured in medium containing no antibiotic (lanes 2, 3, and 6), 4 µg/ml tazobactam (lanes 4 and 7), 4 µg/ml piperacillin (lanes 5 and 8), or 4/4 µg/ml TZP (lane 9) prior to protein sample collection. Molecular mass standards are shown in lane 1 (kilodaltons are indicated to the left of the blot). The estimated molecular mass of Bla_TEM-1_ is 31.5 kDa. This experiment was repeated on independently collected samples and yielded similar results. (B) Determination of Bla_TEM-1_ activity in E. coli 907355 via a nitrocefin hydrolysis assay after growth in the presence or absence of 4/4 µg/ml TZP. ATCC 35218 Bla_TEM-1_ activity is included as a comparison but was not determined (ND) in the presence of TZP due to lack of growth. Units on the *y* axis denote the micromoles of nitrocefin hydrolyzed per milligram of total protein per minute. The values above each bar on the graph represent the means of 3 experiments, and the error bars indicate the standard deviations of the means.

To better understand why E. coli 907355 is resistant to TZP, Bla_TEM-1_ levels were determined by Western immunoblotting following growth in liquid media containing no antibiotic, tazobactam (4 µg/ml), piperacillin (4 µg/ml), or TZP (4/4 µg/ml). A subinhibitory concentration of TZP was utilized, as 907355 does not consistently grow in liquid cultures at higher TZP concentrations despite the fact that it is resistant to TZP in BMD assays. E. coli ATCC 25922 was included as a negative control, as this strain lacks the *bla*_TEM-1_ gene. As expected, Bla_TEM-1_ was not detected in the ATCC 25922 protein sample (Fig. 2A). In contrast, a protein with the estimated molecular mass of Bla_TEM-1_ (31.5 kDa) was detectable in the positive-control strain ATCC 35218 grown in the presence of no antibiotic, tazobactam, or piperacillin.

The Western immunoblotting results showed that E. coli 907355 Bla_TEM-1_ levels varied in response to the antibiotic treatment. In the presence of tazobactam alone, Bla_TEM-1_ levels were lower than under the other growth conditions (Fig. 2A). Quantitation of the 907355 Bla_TEM-1_ band intensities in each lane, which were normalized to total protein levels (data not shown), revealed that tazobactam reduced Bla_TEM-1_ levels by approximately 2.7-fold below those for cells grown without supplementation. This phenomenon was not specific to 907355 Bla_TEM-1_, as it also occurred in ATCC 35218 (Fig. 2A). One possible explanation for this finding is that tazobactam binding destabilizes Bla_TEM-1_, leading to increased degradation of the protein. Alternatively, Bla_TEM-1_ levels may be equal in untreated and tazobactam-treated cells, but tazobactam may partially block the primary antibody binding site during the immunoblotting procedure. We did not investigate this observation further, as the primary goal of this study was to determine the mechanism of Bla_TEM-1_-mediated TZP resistance in E. coli 907355.

The Western immunoblotting also revealed that E. coli Bla_TEM-1_ levels are significantly higher in the presence of TZP in comparison to no antibiotic, tazobactam, or piperacillin (Fig. 2A). Quantitation of the Bla_TEM-1_ band intensities indicated that Bla_TEM-1_ levels were 3.7-fold higher in the presence of TZP versus no antibiotic and 9.9-fold higher in the presence of TZP than with tazobactam alone. Thus, TZP selection considerably increased Bla_TEM-1_ protein levels in E. coli 907355.

To confirm that TZP elevates the amount of Bla_TEM-1_ in E. coli 907355, we measured β-lactamase enzyme activity via a nitrocefin hydrolysis assay after growth in the presence or absence of 4/4 µg/ml TZP. E. coli 907355 Bla_TEM-1_ activity was approximately 2.2-fold higher than that of ATCC 35218 Bla_TEM-1_ in the absence of antibiotic (Fig. 2B). In addition, 907355 Bla_TEM-1_ activity increased by ~3.5-fold in the presence of TZP in comparison to no antibiotic (Fig. 2B). As controls, the experiment was also performed on E. coli 907355 grown in 4 µg/ml piperacillin or tazobactam alone. Piperacillin did not significantly affect Bla_TEM-1_ activity in comparison to no additive, whereas tazobactam inhibited β-lactamase activity, as expected (data not shown). These results are consistent with the finding that TZP increases E. coli 907355 Bla_TEM-1_ production.

Based on previous studies, we suspected that the TZP-induced increase in 907355 Bla_TEM-1_ activity might be due to an increase in *bla*_TEM-1_ gene dosage (13, 15, 22, 23). The copy numbers per cell for E. coli 907355 and ATCC 35218 *bla*_TEM-1_ genes were determined using a SYBR green quantitative PCR (qPCR) assay after bacterial growth in cation-adjusted Mueller-Hinton broth (CAMHB) containing or lacking subinhibitory concentrations of TZP (4/4 µg/ml). As a reference for cell number, qPCR was also performed on the *dxs* gene, which is present in single copy on the E. coli chromosome. The concentration of each *bla*_TEM-1_ product was divided by the concentration of *dxs* in the same sample to determine the relative number of copies of *bla*_TEM-1_ per cell.

**FIG 3:**
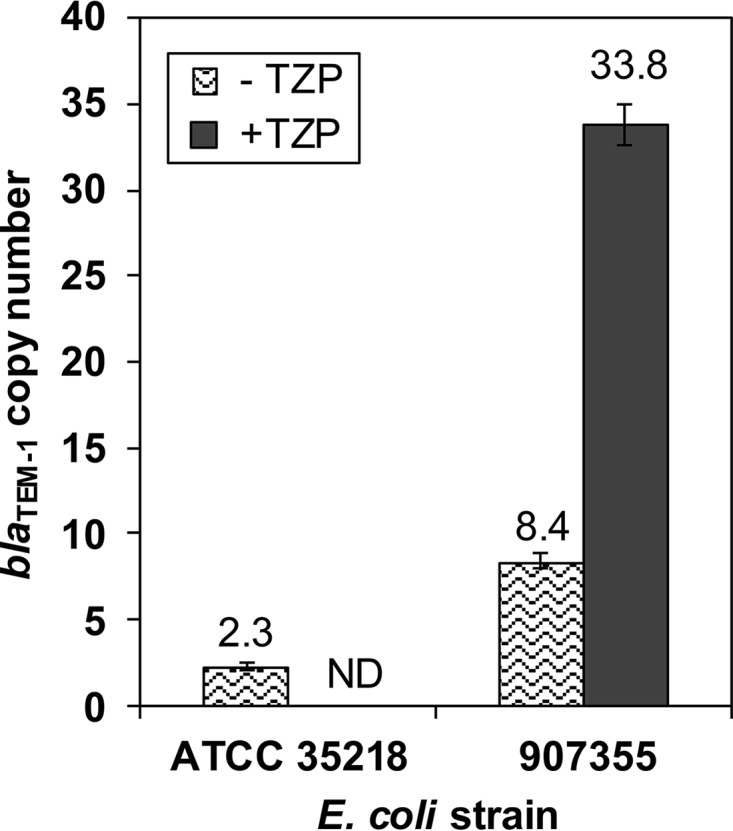
Estimation of bla_TEM-1_ copy number by qPCR. bla_TEM-1_ copy number per cell was measured using a real-time SYBR green qPCR assay after bacterial growth in the absence or presence of 4/4 µg/ml TZP. The copy number was not determined (ND) for ATCC 35218 in the presence of TZP due to lack of growth. The values above each bar on the graph represent the mean results of 3 experiments, and the error bars indicate the standard deviations of the means.

The *bla*_TEM-1_ gene was found to be present at an average copy number of only 2.3 per cell in ATCC 35218 (Fig. 3). In contrast, the 907355 *bla*_TEM-1_ gene copy number per cell rose from 8.4 in the absence of TZP to 33.8 in the presence of TZP. These results suggest that 4/4 µg/ml TZP induces a ~4-fold increase in *bla*_TEM-1_ gene dosage, which is responsible for the elevated Bla_TEM-1_ activity. As controls, piperacillin and tazobactam were tested individually for effects on *bla*_TEM-1_ gene copy number. Neither piperacillin nor tazobactam alone influenced *bla*_TEM-1_ gene dosage (data not shown), indicating that the increase in copy number per cell is due to treatment with a combination of antibiotic and inhibitor.

**FIG 4:**
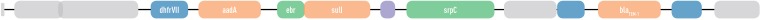
Resistance island containing *bla*_TEM-1_ gene in E. coli 907355. Annotation is based upon the E. coli UMN026 complete genome sequence (10, 27). Genes are shaded according to function: gray, transposon elements and integrase; blue, pseudogenes; orange, antibiotic resistance; green, heavy metal or toxic compound efflux; purple, hypothetical protein.

To further investigate the mechanism of *bla*_TEM-1_ copy number proliferation and genomic changes in E. coli 907355, bacteria grown in absence or presence of TZP were sequenced using Illumina technology. After assembly and annotation of the WGS data, the contig containing *bla*_TEM-1_ was only ~ 2 kb. Therefore, the same samples were sequenced again using the PacBio and MinION platforms, which allow longer raw sequence reads. A draft hybrid assembly constructed from the Illumina and PacBio data, available under BioProject PRJNA431448 on the NCBI website (https://www.ncbi.nlm.nih.gov), revealed that the genome is approximately 5.38 Mb. The *bla*_TEM-1_ gene resides within an approximately 10-kb region, similar to the genomic resistance module (GRM) found in the chromosome of E. coli UMN026 (Fig. 4) (10). Unlike the chromosomal GRM in E. coli UMN026, the 907355 GRM is located on a large plasmid similar to p1ESCUM (additionally called p1ECUMN), which was also first identified in UMN026 (10, 27). MinION sequence data further illuminated the genomic structure and location of the GRM. Examination of these reads confirmed that the GRM in 907355 p1ESCUM interrupts *traI*, which encodes a DNA helicase/relaxase normally required for plasmid conjugative transfer (28).

Previous studies in E. coli have attributed β-lactam/β-lactamase inhibitor or broad-spectrum cephalosporin resistance to the localization of *bla*_TEM-1_ on high-copy-number plasmids (15, 22, 23). To determine whether the observed increase in E. coli 907355 *bla*_TEM-1_ gene dosage is due to p1ESCUM accumulation, we analyzed raw Illumina and PacBio WGS data and calculated approximate copy numbers for *bla*_TEM-1_, *ebr*, and three *tra* genes (*traI*, *traJ*, and *traK*) averaged together (Table 2). Copy numbers were estimated by dividing the sequence coverage for each p1ESCUM gene by the mean coverage for seven single-copy E. coli multilocus sequence type (MLST) genes (*adk*, *fumC*, *gyrB*, *icd*, *mdh*, *purA*, and *recA*). Independent calculations from the Illumina and PacBio WGS data indicated that *bla*_TEM-1_ copy number increased ~4- to 5-fold in the presence of 4/4 µg/ml TZP (Table 2), similar to the results obtained with qPCR (Fig. 3). Raising the TZP concentration to 8/8 µg/ml further augmented the *bla*_TEM-1_ copy number to more than 90 per cell (Table 2). Similarly, the estimated copy number for *ebr*, another gene on the GRM (Fig. 4), increased 4- to 5-fold upon exposure to 4/4 µg/ml TZP and reached 89 copies or more per cell in 8/8 µg/ml TZP (Table 2). In contrast, the average copy number for the three *tra* genes, which are not on the GRM, was ~1 in the absence of antibiotic selection and remained stable upon exposure to TZP (Table 2). Thus, the increase in E. coli 907355 *bla*_TEM-1_ copy number in response to TZP is not simply due to accumulation of the entire p1ESCUM plasmid.

**TABLE 2:** Estimation of *bla*_TEM-1_ copy number based on two WGS technologies

NGS technology	TZP concn (µg/ml)	Mean sequence coverage^a^				Estimated gene copy no.		
		MLST genes	*bla*_TEM-1_ gene	*tra* genes	*ebr* gene	*bla*_TEM-1_ gene	*tra* genes	*ebr* gene
Illumina	None	52.3	554.8	53.0	556.1	10.6	1.0	10.6
	4/4	62.3	3,358.0	94.5	3,218.8	53.9	1.5	51.7
	8/8	30.5	3,450.0	38.9	2,995.7	113.3	1.3	98.3
PacBio	None	113.9	1,363.5	104.2	1,094.0	12.0	0.9	9.6
	4/4	163.4	7,537.5	264.7	6,825.8	46.1	1.6	41.8
	8/8	83.2	7,523.3	160.5	7,410.4	90.4	1.9	89.1

a See text for calculation method.

Based on the copy number analysis of p1ESCUM genes, we hypothesized that the ~10-kb GRM is amplified within the plasmid as a result of TZP selection. In support of this theory, five PacBio reads were identified that contained one full GRM and two partial GRMs on either side (suggesting up to three adjacent GRMs) when 907355 was grown in 8/8 µg/ml TZP. Furthermore, one MinION read was identified that contained three full adjacent GRMs with two partial GRMs on either side (suggesting up to five adjacent GRMs). In both cases, the number of repeats detected was limited by the read lengths provided by the sequencing platform. Reads containing the GRM bordered on both sides by the interrupted *traI* gene were not observed (data not shown). These findings suggest that at higher TZP levels, we were unable to detect the entire amplified region and that the 907355 p1ESCUM plasmid likely contains more than five tandem copies of the GRM.

In some bacteria, inactivation of major porin genes plays a role in *bla*_TEM-1_-mediated β-lactam/β-lactamase inhibitor or cephalosporin resistance (29–32). To determine whether E. coli 907355 major porin genes are intact, we identified homologues of the E. coli K-12 MG1655 *ompF* and *ompC* genes within the 907355 assembled WGS data by using the NCBI BLAST program (https://blast.ncbi.nlm.nih.gov/Blast.cgi). The E. coli 907355 *ompF* and *ompC* genes are predicted to encode full-length proteins that are 99% and 95% identical to their respective homologues in E. coli K-12 MG1655. In addition, the levels of OmpF and OmpC were examined in E. coli 907355 and ATCC 35218 by Western immunoblotting after bacterial growth in CAMHB, the medium used for BMD assays. OmpF expression was barely detectable in both strains, likely due to the relatively high osmolarity of the medium. However, a protein with the predicted molecular mass of OmpF was apparent in both 907355 and ATCC 35218 upon overexposure of the blot (see Fig. S1 in the supplemental material). OmpC, which is highly expressed in CAMHB, was also present at similar levels in 907355 and ATCC 35218 (data not shown). These results show that reduction of OmpF and OmpC expression does not account for 907355 TZP resistance in BMD assays.

10.1128/mBio.00583-18.1 FIG S1  Detection of major porins OmpF and OmpC in E. coli. Download FIG S1, DOC file, 0.1 MB. Copyright © 2018 Schechter et al. 2018 Schechter et al. This is an open-access article distributed under the terms of the Creative Commons Attribution 4.0 International license.

Increased antibiotic efflux might also influence β-lactam/β-lactamase inhibitor resistance in 907355. To examine this possibility, we utilized Phe–Arg–β-naphthylamide (PAβN), a known efflux inhibitor (33, 34). The presence of 50 µM PAβN did not reduce the 907355 TZP MIC in a BMD assay, suggesting that increased efflux may not play a significant role in 907355 TZP resistance (Table 1).

## DISCUSSION

In this study, the mechanism of β-lactam/β-lactamase inhibitor resistance was investigated in E. coli 907355, a clinical isolate containing the *bla*_TEM-1_ gene. Experimental and genomic data support a role for *bla*_TEM-1_ gene amplification, leading to Bla_TEM-1_ hyperproduction, as the primary basis for β-lactam/β-lactamase inhibitor resistance in this isolate. Notably, we found that *bla*_TEM-1_ is located within a ~10-kb region that includes other resistance genes, as well as transposon- and integron-related sequences. Others have termed this element a genomic resistance module, or GRM (10).

In the absence of antibiotic selection, our qPCR and WGS raw read data suggest that the 907355 GRM is present at ~8 to 12 copies per cell (Fig. 3; Table 2). In the presence of 8/8 µg/ml TZP, the GRM copy number per cell increased approximately 10-fold to >90. These results translate to at least an ~800-kb increase in 907355 genomic DNA during TZP treatment and dedication of more than 15% of the E. coli 907355 bacterial genome to antibiotic resistance. Because 907355 TZP resistance is heterogeneous (25), our estimation of GRM levels is likely an average of the various copy numbers present within the population. Therefore, GRM amplification may actually be higher than 10-fold within a proportion of the population.

Multiple examples in the literature document the contributions of tandem gene duplication and amplification to the development of antibiotic resistance (35–38). In fact, two studies have reported that Bla_TEM-1_ hyperproduction may result from tandem amplification of the *bla*_TEM-1_ gene. In one report, Sun et al. engineered a laboratory strain of Salmonella enterica serovar Typhimurium to carry the *bla*_TEM-1_ gene on an F′ plasmid, and then they exposed it to increasing levels of cephalothin or cefaclor by serial passage (32). Several clones with higher MICs contained large regions (36 to 134 kb) of tandemly amplified DNA that included the *bla*_TEM-1_ gene. In another study on a clinical isolate of E. coli, genetic rearrangements and an increase in *bla*_TEM-1_ copy number were observed within a single large, low-copy-number plasmid (13). However, the nature of the amplification was not precisely determined. Thus, our work is significant in that it provides direct evidence for tandem gene amplification of *bla*_TEM-1_ in a clinical isolate.

Our findings lend support to multiple aspects of the model proposed by Sandegren and Andersson, which details a stepwise process for the contribution of gene amplification to the development of antibiotic resistance (35). In the first step, gene duplication and amplification provide initial increased tolerance against an antibiotic (35). Accordingly, our data show that amplification of *bla*_TEM-1_ increases E. coli 907355 tolerance to TZP. Second, tandemly amplified regions are usually genetically unstable and lost in the absence of selection. This theory is supported by the fact that E. coli 907355 *bla*_TEM-1_ copy number and Bla_TEM-1_ levels were much lower when cultures were grown without TZP (Fig. 2 and 3). Third, mutations within the amplified genes or other single-copy genes may allow cells to develop more durable resistance that does not require tandem gene amplification. In fact, several studies have identified decreased membrane permeability as a contributor to β-lactam/β-lactamase inhibitor or cephalosporin resistance in bacteria containing *bla*_TEM-1_ (29–32). Our initial examination of E. coli 907355 membrane permeability revealed that OmpF and OmpC are expressed (see Fig. S1 in the supplemental material) and that PAβN did not significantly reduce the TZP MIC. These results may explain why E. coli 907355 amplifies *bla*_TEM-1_ to such high levels in the presence of TZP. However, it is important to note that E. coli 907355 membrane permeability was not extensively characterized and the isolate may have a deficiency that we did not detect.

In addition to supporting a role for tandem *bla*_TEM-1_ amplification in the development of β-lactam/β-lactamase inhibitor resistance, our work underscores two important implications for clinical microbiology laboratories. First, our data may explain why E. coli isolates that contain only *bla*_TEM-1_ frequently exhibit discordant or heterogeneous behavior when tested by different AST methods for TZP (24, 25). Because the BMD reference method uses a fixed concentration of 4 µg/ml of tazobactam, which binds irreversibly to β-lactamase enzymes, a threshold level of *bla*_TEM-1_ may overcome the available inhibitor. E. coli 907355 may be resistant to TZP in BMD assays, because a cell that amplifies *bla*_TEM-1_ to high enough levels outgrows other cells with fewer copies of *bla*_TEM-1_. In contrast, 907355 may be susceptible to TZP when tested via agar-based methods because cells that express high enough Bla_TEM-1_ levels appear as isolated colonies. Accordingly, increasing the concentration of tazobactam overcame 907355 resistance to TZP in BMD assays (Fig. 1). A similar observation was described previously for other Bla_TEM-1_ hyperproducing strains (39). Taken together, our findings suggest that the *in vitro* resistance of 907355 may represent an artifact of the BMD testing system, which uses a fixed concentration of tazobactam in combination with increasing concentrations of piperacillin. TZP resistance in this case may not translate into clinical resistance of the organism in a patient therapeutic setting, where higher concentrations of tazobactam could be obtained. In support of this theory, neutropenic mice infected with bacteria identified as TZP resistant by BMD, but susceptible to other classes of β-lactams, were successfully treated with humanized exposures of TZP (40, 41).

A second important clinical implication of our work is that *bla*_TEM-1_ gene amplification is difficult to detect by current genotypic antibiotic susceptibility prediction methods. First, clinical isolates are typically subcultured on media without antibiotic selection prior to carrying out both genotypic and phenotypic assays. Because tandemly amplified regions are inherently unstable, they may be lost prior to testing. Second, *bla*_TEM-1_ amplification in E. coli 907355 was not detectable by Illumina sequencing alone, as this method generates short sequencing reads (151 bp) that are not easily assembled in repetitive regions of the genome. Similarly, a recent study employing Illumina sequencing to generate WGS data was unable to accurately predict TZP susceptibility in 13 of the isolates tested (42). Combining Illumina data with longer PacBio reads, which were an average length of ~1.7 kb in our study, allowed a more complete assembly of the E. coli 907355 *bla*_TEM-1_ region. In addition, MinION technology was useful because it produced even longer reads than PacBio, allowing us to detect at least five adjacent copies of the GRM within the genome of the culture treated with 8/8 µg/ml TZP. Finally, classical PCR detection assays for *bla*_TEM_ only reveal that the gene is present and not whether it is tandemly amplified. Comparison of *bla*_TEM_ regions in a variety of clinical isolates resistant to TZP may reveal common sequences that can be utilized for development of PCR assays that detect tandem gene amplification.

Our finding that a clinical isolate of E. coli significantly amplifies *bla*_TEM-1_ in response to TZP raises important questions. For example, how commonly are isolates similar to E. coli 907355 found in clinical settings? Although we cannot answer this question definitively, a preliminary screening of nine other E. coli isolates from our previously published studies revealed that at least four other independent clinical isolates have the ability to substantially amplify *bla*_TEM-1_ (data not shown). We are currently working on characterizing these isolates. Further studies will be required to determine the frequency of similar strains in natural populations.

Another question is whether *bla*_TEM-1_ gene amplification occurs in infected patients during therapy with β-lactam antibiotics or β-lactam/β-lactamase inhibitor combinations. Unfortunately, the antibiotic treatment regimen of the patient from which E. coli 907355 was isolated was not available to us. Furthermore, E. coli 907355 isolates were not collected prior to and over the course of the antibiotic therapy. Future studies in mice might address whether *bla*_TEM-1_ amplification occurs in E. coli 907355 during host infection (40, 41). Recently, McGann et al. reported that *aph1* amplification occurred in Acinetobacter baumanii during tobramycin treatment of an infected patient, leading to antibiotic therapy failure (36). Clinical studies will be required to determine whether extensive *bla*_TEM-1_ amplification contributes to antibiotic therapy failure for β-lactam antibiotics or β-lactam/β-lactamase inhibitor combinations.

## MATERIALS AND METHODS

### Bacterial isolates.

E. coli 907355, a clinical isolate received from Austria, has been described previously (24, 25). E. coli ATCC 25922 (*bla*_TEM-1_ negative) and/or ATCC 35218 (*bla*_TEM-1_ positive) quality control organisms recommended by CLSI for AST were used as comparators in experiments. Although ATCC 35218 produces Bla_TEM-1_, it is susceptible to β-lactam/inhibitor combinations, including TZP.

### AST.

MICs were determined by BMD according to CLSI standard methods, using CAMHB supplemented with appropriate ranges of β-lactam antibiotics (26, 43). β-Lactamase inhibitors were included at the recommended fixed concentrations, except that the concentration of tazobactam varied between 2 and 64 µg/ml for the experiment in Fig. 1 (26). Resistance or susceptibility was interpreted using CLSI breakpoints (26). When skipped wells were encountered during reading of the MIC, CLSI recommendations were followed for interpretation.

### Bacterial growth and sample collection.

For all Bla_TEM-1_ protein characterization assays and *bla*_TEM-1_ qPCR studies, bacteria were cultured in CAMHB with no antibiotic, 4 µg/ml tazobactam, 4 µg/ml piperacillin, and/or 4/4 µg/ml TZP. Cultures were inoculated with a 1:300 dilution of a 0.5 McFarland suspension and incubated overnight at 35°C without agitation. After 18 to 20 h, cells were harvested by centrifugation. For Western immunoblotting, equal amounts of cells (~1.5 ml of each culture) were pelleted based on readings of the optical density at 600 nm. Pellets were then suspended in 100 µl 2× Laemmli sample buffer (Bio-Rad) and frozen at −20°C. For enzyme activity and PCR experiments, pellets (from 1-ml culture aliquots) were suspended in 0.1 M sodium phosphate buffer, pH 7.0.

For WGS analysis, E. coli 907355 bacteria were cultured overnight on Mueller-Hinton agar (MHA) plates supplemented with 4/4 µg/ml, 8/8 µg/ml, or no TZP. Approximately 10 isolated colonies were then inoculated into CAMHB containing the same concentrations of antibiotics as the MHA plates. Cultures were grown overnight at 35°C with agitation and pelleted by centrifugation. Pellets were stored at −20°C prior to DNA isolation.

### Isoelectric focusing and Western immunoblotting.

IEF was performed using pH 3 to 10 precast IEF protein gels (Novex) and the XCell SureLock Mini-Cell (Life Technologies, Inc.) per the manufacturer’s instructions. β-Lactamase proteins were detected with 1 mM nitrocefin (Remel), using known enzymes as standards.

For Western immunoblotting, samples were boiled for 5 min and loaded (12 µl) onto a Mini-Protean TGX Stain-Free gel (Bio-Rad). Precision Plus protein standards (Bio-Rad) were run simultaneously to estimate molecular weight. Following separation by electrophoresis in a Protean Tetra cell (Bio-Rad), proteins were transferred onto a polyvinylidene difluoride membrane by using the Trans-Blot Turbo system (Bio-Rad). Bla_TEM-1_ was detected using a standard Western immunoblotting procedure with the Clarity Western ECL substrate (Bio-Rad), as instructed by the manufacturer. Primary anti-β-lactamase 8A5.A10 mouse monoclonal antibodies (Santa Cruz Biotechnology) were used at a 1:200 dilution, and secondary goat anti-mouse IgG–horseradish peroxidase conjugate antibodies (Bio-Rad) were used at a 1∶3,000 dilution. Blots were developed on a ChemiDoc MP imager with Image Lab v5.2 software (Bio-Rad). Total protein on the blot was visualized using the Stain-Free protocol, whereas chemiluminescent signals were captured using the Chemi-Hi resolution protocol. Relative levels of Bla_TEM-1_ in E. coli 907355 samples were calculated by the Image Lab software, which quantitated the intensities of the chemiluminescent Bla_TEM-1_ bands and normalized them to total protein.

### β-Lactamase enzyme activity assays.

Lysates for β-lactamase enzyme studies were prepared using glass beads as previously described (44). Briefly, cell suspensions in phosphate buffer were transferred to microcentrifuge tubes containing about 0.25 g of 100-µm glass beads. The tubes were vortexed at full speed with a bead beater attachment at 4°C for 10 min and then centrifuged at 16,000 × *g* at 4°C for 15 min. Supernatants were transferred to clean tubes and stored at −20°C until testing. β-Lactamase activity assays were carried out in triplicate in an Infinite M200 Pro microplate reader (Tecan) at 25°C using 100 µM nitrocefin and 5 µl of each crude glass bead lysate, as previously described (11, 45). Enzyme rates were normalized to the total protein present in each lysate, which was determined with a bicinchoninic acid assay kit (Thermo Scientific) per the manufacturer’s instructions.

### Determination of *bla*_TEM-1_ copy number by PCR.

Lysates for *bla*_TEM-1_ gene copy number analysis were prepared by boiling bacterial suspensions for 10 min, followed by storage at −20°C prior to testing (46). Real-time qPCR was performed in a LightCycler 1.5 system (Roche) with SYBR green I (Roche) and primers that hybridize to the target gene *bla*_TEM-1_ or the single-copy chromosomal reference gene, *dxs* (d-1-deoxyxylulose 5-phosphate synthase), using a method similar to that reported by Lee et. al. (47). However, the reverse primer for *dxs* was moved downstream 1 nucleotide, based on mismatches detected for other E. coli *dxs* sequences in GenBank. Native *Taq* DNA polymerase isolated from Thermus aquaticus (Invitrogen/Thermo Fisher Scientific) was utilized in all reaction mixtures due to the possible contamination of recombinant *Taq* preparations with *bla*_TEM-1_ DNA. Absolute quantification analysis was performed using LightCycler software v3.5.3 with the automated method. An internal calibrator of 1.5 × 10^5^ copies of pBR322/μl spiked into 1.5 × 10^5^ CFU/µl of DH5α was included in each run to control for PCR efficiency and was used to normalize the target and reference results from each sample. To determine the concentrations of *bla*_TEM-1_ or *dxs* in each sample, normalized crossing point (Cp) values were compared to a standard curve generated by performing qPCR on known quantities of each target. The *dxs* standard curve was generated by performing qPCR on DH5α lysates made from samples with a range of established CFU per microliter (4.7 × 10^3^ CFU/µl up to 6 × 10^5^ CFU/µl). The *bla*_TEM-1_ standard curve was generated by qPCR on samples containing a range of pBR322 DNA concentrations (1.3 × 10^3^ plasmids/µl up to 1.0 × 10^8^ plasmids/µl) spiked into DH5α. The number of double-stranded copies per microliter of *bla*_TEM-1_ was then divided by the number of double-stranded copies of *dxs* per microliter to determine the relative number of copies of *bla*_TEM-1_ per cell.

### Bacterial genome sequencing and data interpretation.

E. coli 907355 genomic DNA was isolated from cultures containing 0, 4/4 µg/ml, or 8/8 µg/ml TZP by using the DNeasy blood and tissue kit (Qiagen) according to the manufacturer’s instructions. A whole-genome shotgun library was then constructed from each sample by using a TruSeq Nano DNA High-Throughput Library Prep kit (Illumina) according to Illumina’s standardized protocol. Dual indexed paired-end libraries with an average insert size of 800 bp were made by shearing 200 ng genomic DNA with a Q800R sonicator system (QSonica) in a total volume of 50 µl 1× Tris-EDTA buffer. The sheared DNA was cleaned and size selected using sample purification beads provided in the library preparation kit. Universal adapters for paired-end sequencing and indexing were then added according to an optimized protocol from Illumina. The indexed libraries were pooled and sequenced on a HiSeq 2500 system (Illumina) with a target of 100× coverage per genome.

PacBio sequencing libraries were prepared using an SMRTbell Template Prep kit 1.0 (Pacific Biosciences) according to the manufacturer’s standard protocol. Genomic DNA was extracted using an extraction kit (Qiagen) and 1 µg of DNA was used as the template to prepare SMRTbell libraries. DNA was sheared using a Covaris g-TUBEs apparatus at 4,000 rpm for 3 to 4 min and cleaned using 0.75× AMPure XP beads (Beckman Coulter, Inc.) to size select the desired range of fragments and remove all DNA fragments of <200 bp. SMRTbell-adapted libraries were sequenced on one SMRT cell, using P6C4v2 chemistry. Output files were processed and assembled into CCS reads by using PacBio RSII SMRT portal software (v2.3.0) default settings with minimum passes at three and minimum predicted accuracy of 0.9.

MinION sequencing libraries were prepared from unsheared genomic DNA using the SQK-MAP005 2D library preparation kit (Oxford Nanopore Technologies) and the EXP-LWI001 low-input expansion kit (Oxford Nanopore Technologies) when less than 1 µg of DNA was available. To ensure minimal shearing during the library preparation process, the general procedure provided by Oxford Nanopore Technologies was adapted to use of wide-bore pipette tips, as well as a HulaMixer (Thermo Fisher Scientific) in place of vortexing. Libraries were sequenced using R7.3 chemistry, and poretools was used to extract fastq files from the native HDF5 format (48).

A hybrid *de novo* assembly of PacBio and Illumina reads was generated using SPAdes 3.9 with the “multiple-kmer” and “careful” options (49). All assembled contigs were annotated using the automated annotation software Prokka v1.11 (50). A BLAST search against NCBI GenBank identified the closest homologous E. coli completed genome, and then a subsequent BLAST against this completed genome was performed to manually refine annotation of the *bla*_TEM-1_-containing element (51).

### Determination of gene copy number by analysis of WGS data.

Copy numbers of individual genes were determined by calculating ratios of the coverage of the gene of interest to the mean coverage of seven E. coli MLST genes (http://mlst.warwick.ac.uk/mlst/dbs/Ecoli). These MLST genes are involved in housekeeping functions and are known to be present in single copy within the genome. The copy number analysis was performed separately for the Illumina and PacBio sequencing data. To estimate the copy number of the p1ESCUM-like plasmid, the copy number of the *traI*, *traJ*, and *traK* genes was averaged and compared to the mean MLST gene copy number. Read mapping and coverage estimation were performed by using BWA v0.77 and SAMtools v0.1.19 (52, 53).

### Efflux inhibitor assay.

An assay using PAβN (Sigma-Aldrich) was performed as previously described, except TZP was used (54). A ≥4-fold decrease in TZP MIC in the presence of PAβN was considered a significant decrease.

### Outer membrane protein detection.

Samples for outer membrane protein detection were prepared by diluting a 2.0 McFarland suspension 1:200 in 5 ml CAHMB. Cultures were grown to mid-log phase (~2.5 to 3.0 McFarland), and equal volumes of cells based on McFarland readings (~1 ml) were pelleted in a microcentrifuge. The pellets were then suspended in 100 µl 2× Laemmli sample buffer and frozen at −20°C.

Outer membrane proteins were detected according to the Western immunoblotting procedure described above, except using different antibodies. Primary antibodies, either anti-OmpF or anti-OmpC polyclonal IgG (Biorbyt), were used at a 1∶1,000 dilution. Secondary goat anti-rabbit IgG–horseradish peroxidase conjugate antibodies (Bio-Rad) were used at a 1∶3,000 dilution.

### Accession number(s).

All sequence and assembly data are accessible through NCBI BioProject PRJNA431448.
