# Supplementary material for: Extensive Gene Amplification as a Mechanism for Piperacillin-Tazobactam Resistance in Escherichia coli
Source: mBio. 2018 Apr 24;9(2):e00583-18. doi: 10.1128/mBio.00583-18 (PMC5915731; doi:10.1128/mBio.00583-18)
Supplement: FIG S1 [file mbo002183838sf01.doc]

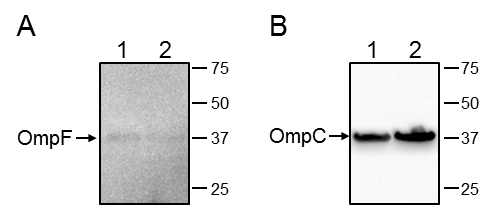


**Figure S1. Detection of major porins OmpF and OmpC in *E. coli*.** Western analysis was performed on protein extracts prepared from *E. coli* strains 1) ATCC 35218 and 2) 907355 using antibodies against A) OmpF, or B) OmpC. The positions of molecular weight markers (in kilodaltons, or kDa) are shown to the left of the blot. The predicted molecular weights for OmpF and OmpC are 39.3 kDa and 41.2 kDa, respectively.
